# Age, body weight and ovarian function affect oocyte size and morphology in non-PCOS patients undergoing intracytoplasmic sperm injection (ICSI)

**DOI:** 10.1371/journal.pone.0222390

**Published:** 2019-10-24

**Authors:** A. Weghofer, V. A. Kushnir, S. K. Darmon, H. Jafri, E. Lazzaroni-Tealdi, L. Zhang, D. F. Albertini, D. H. Barad, N. Gleicher

**Affiliations:** 1 Department of Obstetrics and Gynecology, Medical University of Vienna, Waehringer, Guertel, Vienna, Austria; 2 The Center for Human Reproduction, New York, New York, United States of America; 3 Department of Obstetrics and Gynecology, Wake Forest University, Winston-Salem, North Carolina, United States of America; 4 Stem Cell Biology and Molecular Embryology Laboratory, The Rockefeller University, New York, New York, United States of America; 5 The Foundation for Reproductive Medicine, New York, N.Y., United States of America; Peking University Third Hospital, CHINA

## Abstract

The size of oocytes was previously reported to be smaller in obese women with polycystic ovary syndrome (PCOS). In the present prospective cohort study, we sought to determine whether oocyte size and morphology are associated with patient characteristics in non-PCOS women. Oocyte and oolemmal diameter were measured, enlarged perivitelline space (PVS) and ooplasmic granulation were assessed in 308 MII oocytes from 77 IVF/ICSI couples. Statistical analysis was undertaken using SAS version 9.4 (SAS institute Inc., USA). Continuous values are presented as mean ± SD and compared using a two-sample t-test or Mann-Whitney U test as appropriate. Categorical parameters are presented as proportions and compared using a Fisher exact test. Logistic and linear regression models were used to control for the effect of age for categorical and continuous variables respectively. P-value < 0.05 was considered statistically significant. Patients presented with a mean age of 40.3±5.0 years, had a BMI of 25.1±6.1 kg/m^2^, median AMH levels of 0.6 ng/ml and produced a median of 4 oocytes. Mean total oocyte diameter was 163.2±7.4 μm (range 145.8–182.1 μm), while oolemmal diameter was 109.4±4.1 μm (range 98.5–122.3 μm). After adjusting for age and ovarian reserve increasing BMI was associated with decreased total oocyte diameter (p<0.05). Total oocyte diameter was also inversely associated with AMH levels (p = 0.03) and oocyte yield (p = 0.04). In contrast to total oocyte diameter, oolemmal diameter was not related to patient characteristics. Younger women and those with large oocyte yields demonstrated fewer oocytes with ooplasmic granulation (p<0.05 and p = 0.01). After adjustments for age, ooplasmic granulation was also less frequently observed in oocytes from women with higher AMH (p = 0.03) and increasing BMI (p<0.01). Fertilization was more likely in oocytes with larger oolemmal diameter (p = 0.008). Embryos from oocytes with larger total and ooplasmic diameters were more likely to be transferred or frozen (p = 0.004 and p = 0.01). In non-PCOS infertile women, BMI and ovarian function relate to total oocyte diameter. These results expand on previously observed associations between oocyte size and BMI in women with PCOS. They indicate the importance of detailed oocyte assessments, which may aid the currently used criteria for embryo selection and help to better understand how oocyte status is associated with later embryo development.

## Introduction

In the course of assisted reproduction, the embryo’s competence to properly develop is considered crucial for achieving pregnancy [1]. Most in vitro fertilization (IVF) programs select embryos suitable for transfer based on embryo morphology [2] and more recently increasingly using 24 chromosome genetic screening [3, 4]. Research on cumulative pregnancy rates after fresh and frozen embryo transfers demonstrates, however, that currently utilized embryo grading alone or in combination with genetic screening has limited potential to identify embryos with maximum implantation potential [5]. In an attempt to further improve embryo selection by continuous visualization of embryonic development, time-lapse imaging systems have been introduced over recent years, but failed to improve live birth rates [6, 7]. Similarly, PGT while increasing pregnancy rates per transfer, has failed to provide improved cumulative pregnancy and live birth rates [8].

These findings raise the question whether oocyte morphology may add to our current understanding of embryo development and implantation potential. Morphokinetic studies support this hypothesis demonstrating embryo quality to primarily relate to oocyte, rather than to sperm parameters [9, 10]. Excellent live birth rates achieved in oocyte donation cycles–irrespective of paternal age–underscore these observations [11].

Other research groups and we have reported benefits of more detailed oocyte assessments in predicting embryo development and pregnancy potential [12, 13]. Very large oocyte yields are predominantly observed in women with polycystic ovary syndrome (PCOS). Oocyte morphokinetics may, therefore be especially visible in patients with PCOS: For example, Marquard and co-workers in PCOS demonstrated associations between oocyte sizes and body weight [14]. Atzmon et al, found oocytes from obese women (excluding PCOS) were significantly smaller than those from normal weight women [15]. Their findings are supported by animal studies showing smaller oocytes, maturation delays and increased granulosa cell apoptosis in diabetic, insulin-resistant and obese mice [16].

Animal studies go even further and demonstrate associations between oocyte diameter and meiotic competence irrespective of the metabolic disturbances of PCOS [17, 18]. And, knockout studies in mice have identified regulators of oocyte size as oocyte specific genes, with corresponding consequences on follicle growth [19, 20]. These reports suggest that several patient-specific characteristics could influence oocyte diameter and, consequently, the developmental ability of gametes and zygotes. Studies evaluating such effects in humans are, however, scarce.

To investigate whether female age, body mass index (BMI), Anti-Müllerian hormone (AMH) levels and oocyte yield relate to oocyte diameter and morphology in non-PCOS patients was the subject of this study.

## Materials and methods

This observational study prospectively investigated 308 mature (MII) oocytes from 77 women who consecutively underwent controlled ovarian hyperstimulation, intracytoplasmatic sperm injection (ICSI) and morphokinetic evaluation of oocytes and embryos at the Center for Human Reproduction (CHR) in New York City between April and October 2017. Patients were not eligible for enrollment if they presented with a diagnosis of PCOS according to the Rotterdam criteria [21] or were scheduled for egg freezing. Inclusion also precluded oocyte fertilization via IVF instead of ICSI, as oocyte assessment and imaging required that oocytes were denuded. Male partners in the study group all had normal semen analysis. Cancelled cycles and those without oocytes at retrieval were excluded from the analysis.

At initial presentation, all women underwent the center’s routine work up, including medical history, vaginal ultrasound and ovarian reserve testing with FSH and AMH on cycle days 2/3. There was no AMH cutoff for inclusion in this cohort. Patients underwent controlled ovarian hyperstimulation with GnRH agonists and FSH/hMG in a 3:1 ratio according to ovarian function. Thirty-six hours after ovulation induction with 10,000 IU of hCG (various manufactures), oocyte retrieval was performed.

Oocyte preparation, imaging, measurements, fertilization and embryo assessment were performed as previously reported [13]. After retrieval oocytes were incubated with Human Tubal Fluid (HTF) media (LifeGlobal, LLC, Gilford, CT) with 10% of Human Serum Albumin (HSA, LifeGlobal, LLC, Gilford, CT) for a minimum of two hours. Oocytes were then stripped of cumulus cells with 80 IU/ml hyaluronydase (LifeGlobal, LLC, Gilford, CT) and mechanically denuded. For intracytoplasmic sperm injection (ICSI) mature oocytes were placed into an ICSI dish with 25ul drops of HTF with HEPES, with 10% of HSA added under mineral oil (LifeGlobal, LLC, Gilford, CT). Each drop was labeled numerically. Immediately before ICSI digital imaging of the oocytes was performed using INFINITY software, Lumenera Corp, Ottawa, Ontario, Canada. All images were captured using an Olympus IX71 inverted microscope and a 20X Planapo objective with a Tokai heating stage. A stage micrometer was used to calibrate final magnifications for the 20X objective from which all measurements were made. Total oocyte diameter was measured as the maximum diameter of zona pellucida enclosed oocytes. Oolemmal diameter was measured as the maximum diameter of the oolemma. Perivitelline space (PVS) and ooplasmic granulation were assessed. After ICSI, oocytes were cultured individually under mineral oil in labeled drops of blastocyst media (LifeGlobal, LLC, Gilford, CT) with 10% HSA.

About 18 hours after ICSI presence or absence of pronuclei (PN) was evaluated. Culture dishes with the zygotes were then incubated uninterruptedly until day three. Routinely, day 3 embryo assessments were performed. Blastomere number and symmetry, granularity, and fragmentation were assessed. Embryos were graded ad follows: Excellent or Grade 5, reached at least 8 cells with <5% fragmentation; Good or Grade 4, reached 6–8 cells with 5% fragmentation; Average or Grade 3, 6–8 cells with 5–20% fragmentation; Poor or Grade 2, reached 4–8 cells with 20–40% fragmentation; Very poor or Grade 1, 4–8 cells, with >40% fragmentation. Embryo transfer was routinely scheduled on day-3 after fertilization at cleavage-stage.

Statistical analysis was undertaken using SAS version 9.4 (SAS institute Inc., USA). Continuous values are presented as mean ± SD and compared using a two-sample t-test or as median and interquartile range M(IQR) in the case of non-normality and compared using the Mann-Whitney U test. Categorical parameters are presented as proportions and compared using a Fisher exact test. Logistic and linear regression models were used to control for the effect of age for categorical and continuous variables respectively. Number of oocytes retrieved was log converted to account for non-normality and compared using a Pearson’s correlation test. Because multiple oocytes were retrieved from each patient, analysis of oocyte characteristics and patient characteristics was performed using repeated measures models within patient’s oocyte cohort. P-value < 0.05 was considered statistically significant.

Patients at our center sign an informed consent at initial consultation, which allows for use of their anonymized electronic medical records and, where needed, of their paper records, if the patient’s identity remains protected and the medical record remains confidential. Since here presented data only involved review of the center’s anonymized electronic research database also used to report the center’s annual IVF outcomes to national registries, these conditions were met. The Instituational Review Board (IRB) of The Center for Human Reproduction approved such medical record studies by expedited review.

## Results

Patient characteristics, cycle parameters and details on oocyte and embryo development are displayed in Table 1.

**Table 1:** Patient characteristics and cycle parameters and details on oocyte and embryo development are displayed in Table 1.

	Mean		SD	Median	IQR	Q1	Q3
**Female age (years)**	40.3	±	5.0	41	7	37	44
**BMI (kg/m2)**	25.1	±	6.1	22.9	7	21.3	28.2
**AMH (ng/mL)**	1.2	±	1.7	0.6	1	0.3	1.3
**Total gonadotropins used (IU)**	4135	±	1618	4200	1875	3375	5250
**Oocytes retrieved (n)**	6.9			4	6	2	8
**High-quality embryos (n)**	2.6	±	2.6	2	2	1	3
**Embryos transferred (n)**	2.0	±	1.1	2	2	1	3

Values are presented as means ± standard deviation (SD) and as median and interquartile range (IQR).

Mean total oocyte diameter was 163.2±7.4 μm (range 145.8–182.1 μm), while oolemmal diameter was 109.4±4.1 μm (range 98.5–122.3 μm). Eighteen patients had supernumerary embryos available for cryopreservation.

Increasing age was associated with increased total oocyte diameter (P = 0.04). However, age was not a significant predictor of total oocyte diameter when combined in models with other indicators of ovarian reserve (AMH and Oocyte Yield). Interestingly, in these models increased age was associated with increased oocyte diameter while indicators of increasing ovarian reserve (AMH levels (p = 0.03) and oocyte yield (p = 0.04)) were associated with decreased total oocyte diameter. Models combining these factors were not statistically significant suggesting that, as might be expected, they share common variance. The effect estimates of age, AMH and oocyte yield for total oocyte diameter were stable in the various models and each remained significant when combined with BMI alone. Age was not significantly associated with changes in the oolemmal diameter, PVS or appearance of ooplasmic granulation (Table 2).

**Table 2:** Association of oocyte and patient characteristics: Repeated measure methods per patient*.

	Total oocyte diameter		Oolemmal diameter		Perivitelline space		Granular cytoplasm	
	Estimate	p-value	Estimate	p-value	OR	p-value	OR	p-value
*Age*	0.21	0.04	0.05	0.36	1.02	0.55	1.02	<0.05
*Age*	0.12	0.34	0.06	0.37	0.98	0.71	1.01	0.47
*Oocyte yield*	-0.75	0.29	0.07	0.83	0.76	0.11	0.89	0.04
								
*BMI*	-0.19	0.09	-0.03	0.53	1.03	0.40	0.95	< 0.01
*BMI*	-0.16	0.08	-0.03	0.62	1.03	0.33	0.96	< 0.01
*Age*	0.19	0.03	0.05	0.37	1.39	0.51	1.01	0.12
*BMI*	-0.19	0.04	-0.03	0.57	1.02	0.44	1.05	0.02
*Oocyte yield*	-1.15	0.01	-0.14	0.58	0.79	0.07	1.49	<0.001
*BMI*	-0.18	0.07	-0.03	0.56	1.00	0.50	1.04	0.08
*AMH*	-0.78	0.01	-0.16	0.42	1.04	0.01	1.32	<0.001
*BMI*	-0.18	<0.05	-0.02	0.65	1.00	0.50	1.05	0.02
*Age*	0.10	0.42	0.06	0.36	1.00	0.78	0.99	0.73
*Oocyte yield*	-0.43	0.68	0.40	0.44	0.99	0.87	1.31	0.35
*AMH*	-0.31	0.62	-0.28	0.41	1.04	0.32	1.07	0.69
								
*AMH*	-0.80	0.03	-0.16	0.43	0.82	0.02	1.28	0.01
*AMH*	-0.49	0.25	-0.07	0.72	0.79	0.02	0.93	0.03
*Age*	0.15	0.22	0.04	0.47	0.98	0.63	1.01	0.47
*AMH*	-0.27	0.67	-0.25	0.45	0.78	0.26	1.05	0.79
*Oocyte yield*	-0.88	0.37	0.15	0.74	1.40	0.82	1.31	0.29
*AMH*	-0.33	0.60	-0.28	0.41	1.04	0.36	1.07	0.74
*Age*	0.13	0.33	0.06	0.34	1.00	0.85	0.99	0.73
*Oocyte yield*	-0.36	0.73	0.41	0.44	1.00	0.98	1.23	0.52
								
*Oocyte yield*	-1.19	0.04	-0.15	0.58	0.79	<0.05	0.87	0.01
*Oocyte yield*	-0.75	0.29	0.07	0.83	0.76	0.11	0.89	0.04
*Age*	0.12	0.34	0.06	0.37	0.98	0.71	1.01	0.47

* Both AMH and oocyte yield were log converted in these analyses

BMI was associated with smaller total oocyte diameter only in models that also adjusted for factors reflecting ovarian reserve. After adjusting for ovarian reserve increasing BMI was associated with decreased total oocyte diameter (p<0.05).

None of the factors studied were associated with significant change in oolemmal diameter. AMH and oocyte yield were associated with decreased PVS, as might be expected since they were also associated with decreased total oocyte diameter.

Decreased ooplasmic granulation was significantly associated with increased oocyte yield (p = 0.01) and with increasing BMI (p<0.01). This significant association of oocyte yield was maintained in models adjusted for age (p<0.01), however the effect of increasing BMI was reversed in models adjusted for age and ovarian reserve.

As might be expected, higher oocyte yield was associated with a decreased proportion of oocyte with granulation (p = 0.01) and this association persisted even after adjustment for age. Higher AMH levels were associated with increased ooplasmic granulation (p = 0.01) but when adjusted for age this association was reversed (p = 0.03).

Fertilization, embryo grade and number of high-quality embryos transferred or cryopreserved were all more likely observed in oocytes with larger total oocyte and oolemmal diameter (Table 3). However, the presence of granular cytoplasm and increased perivitelline space was not associated with fertilization, embryo grade, or embryos transferred or cryopreserved (S1 and S2 Tables).

**Table 3:** Effect of oocyte characteristics on embryonic development.

	Fertilization		p	Embryo Grade		p	Transferred/Cryo		p
	No (N = 104)	Yes (N = 204)		Fair/Poor (N = 97)	Good (N = 120)		No (N = 129)	Yes (N = 179)	
***Total Oocyte Diameter***	161.4 ± 7.6	162.6 ± 7.2	0.20	161.3 ± 7.5	163.8 ± 6.5	0.009	160.8 ± 7.2	163.2 ± 7.2	0.004
***Oolemmal Diameter***	108.4 ± 4.5	109.8 ± 4.1	0.008	109.1 ± 3.9	110.0 ± 4.2	0.09	108.6 ± 4.2	109.8 ± 4.3	0.01

In models adjusted for age (Table 3) both total oocyte and oolemmal diameter were associated with increased fertilization. All four oocyte parameters were associated in all adjusted models with increased numbers of embryos suitable for transfer or cryopreservation. Interestingly the presence of granular cytoplasm was associated with increased odds of fertilization and number of embryos transferred or cryopreserved (Table 4).

**Table 4:** Logistic regression models on the impact of oocyte parameters on fertilization and embryo development.

	OR	95% C.L.		p	aOR	95% C.L.		p	aOR	95% C.L.		p
***Fertilization***												
***Age***	0.971	0.944	1.000	0.047	0.968	0.941	0.997	0.031	0.975	0.947	1.004	0.088
***Total Oocyte Diameter***	1.034	1.012	1.057	0.003	1.036	1.013	1.058	0.002				
***Oolemmal Diameter***	1.067	1.030	1.106	0.000					1.065	1.028	1.104	0.001
***Number of embryos transferred or cryopreserved***												
***Age***	1.042	1.012	1.073	0.006	1.032	1.002	1.064	0.039	1.038	1.007	1.071	0.016
***Total Oocyte Diameter***	1.037	1.015	1.060	0.001	1.032	1.009	1.055	0.006				
***Oolemmal Diameter***	1.048	1.010	1.090	0.009					1.059	1.021	1.100	0.002
***Granular Cytoplasm***	1.910	1.343	2.717	0.000	1.667	1.158	2.399	0.006	1.587	1.100	2.292	0.014
***Perivitelline Space***	1.951	1.150	3.310	0.013					2.151	1.233	3.755	0.007

Of the 77 patients in this cohort 10 became pregnant and only 4 experienced a live birth. We observed no association of total oocyte diameter or oolemmal diameters with pregnancy or live birth.

## Discussion

Our data demonstrate that the factors associated with oocyte diameters interact in ways that may not seem intuitively obvious. Both increasing BMI and increased oocyte yield and AMH are associated with decreased oocyte diameter, mostly due to a decrease in perivitelline space in the presence of an unchanging oolemmal diameter. Thus, it seems surprising that in spite of the above associations, increasing total oocyte diameter and oolemmal diameter are associated with increased fertilization, embryo grade and numbers of embryos transferred or cryopreserved, and that the effect of increasing diameters on improved fertilization and number of embryos transferred or cryopreserved persist even after adjustment for other factors.

When evaluating these seemingly disparate effects we must be aware of some characteristics of the cohorts of oocytes being analyzed. Within each patient there is considerable variance in oocyte diameters. This variance nominally increases as patients age with younger patients clustering closer to a median. Older patients have more high and low outliers. Patients with higher levels of ovarian reserve have more oocytes and thus a greater degree of variance in oocyte size, some of which have small diameter and decrease the average diameter of the cohort for each patient. Thus, oocytes with diameters closer to the average mean diameter have better potential.

The patient cohort studied was an older group of patients with evidence of diminished ovarian reserve, thus these observations may not be applicable to other younger patients with normal ovarian reserve. Still, it is informative to learn that in the presence of diminished ovarian reserve oocyte parameters can be somewhat predictive of embryo outcomes. It is not surprising that the oocyte parameters had little impact on pregnancy or live birth since all embryos transferred were derived from the more favorable oocytes in each of the patient’s oocyte cohorts.

These results expand on previously observed interrelationships between oocyte size and BMI in women with PCOS [14]. The impact of oocyte size measurements further translates to fertilization potential and embryo quality. At first sight it may appear surprising that major contributors of procreative potential like female age, AMH, BMI and oocyte yield translate to the morphology of human oocytes beyond maturity. However, there is a growing impression in human ARTs that events occurring during oogenesis within the follicle have a major impact on embryonic competence. During follicular growth and maturation cumulus cells provide substrates and mediate the oocyte’s metabolism up to and through meiotic maturation [22]. Once the oocyte transits from the non-growing quiescence of the primordial follicle pool towards follicular recruitment and selection, it demands gene activation, protein synthesis and hyperplasia of organelles to initiate and sustain growth and hypertrophy [23]. Variable oocyte diameters in women of advanced reproductive age, likely, reflect impaired protein synthesis and an oocyte’s decreased capacity to maintain adequate cell volume [24].

Animal models further suggest a relationship between oocyte size and apoptosis. In most species genetically programmed cell death occurs to properly nourished germ cells while they develop. Andux and Ellis provide evidence for oocyte quality-related defects in apoptosis that hamper ageing nematodes to provide sufficient resources to nurture oocytes to full size [25]. These findings are also supported by reports of altered granulosa cell gene expression in aging females [26] and further point out the importance for female procreation of the synergistic interplay between oocytes and their surrounding granulosa cells. Factors originating in the oocyte influence the course of follicle development and directly or indirectly regulate the final size and oocyte can achieve [27]. Thus, granulosa cells and oocytes establish a follicular microenvironment that provides the oocyte with energy, synchronizes oogenesis with folliculogenesis, and engages in molecular signaling [26].

The above results may also contribute to our understanding of the significance of ooplasmic granulation. Ooplasmic granulation appeared to increase with decreased oocyte yield, lower BMI and lower AMH (when adjusted for age). How granulation comes about is poorly understood but is likely to be due to organelle or protein aggregation, both signs of cellular aging [28]. We observed no association of ooplasmic granulation with oocyte diameters, proportion of fertilization or high-grade embryos. However, in models adjusted for age and oocyte size parameters the presence of ooplasmic granulation was associated with an increase in the number of oocytes transferred or cryopreserved, suggesting that granulation may not be a negative predictor of oocyte quality.

Recent microanalyses demonstrated that alterations in granulosa cell gene expression are not exclusively age-related. Dysregulation of granulosa cell genes that are engaged in oxidative stress reactions, in lipid metabolism and insulin signaling have also been reported in women suffering from PCOS [29] and could perhaps be factors affected non-PCOS women as well. Keefe and colleagues have for some time suspected repetitive periods of oxidative stress to be causally related to DNA mutations of the oocyte’s mitochondrial genome [30], resulting in oocytes’ declining ability to deal with exposure to free radicals, thus contributing to oocyte aging [31, 32]. Adverse effects of oxidative stress, furthermore, have also been held responsible for previously noted associations between smaller oocyte diameter and obesity as well as PCOS [14].

Increasing evidence also suggests that intrafollicular alterations, at least partially, may account for impaired reproductive outcomes in association with IVF in overweight and obese women, characterized by intrafollicular changes in steroidogenesis, metabolism and inflammation [33]. In agreement with these observations and our findings on smaller oocyte diameter with increasing BMI, Leary and associates reported smaller oocytes in overweight and obese patients. Their embryos were not only less likely to reach blastocyst stage, but also experienced decreased glucose consumption, altered amino acid metabolism as well as increased triglyceride levels [34].

## Conclusions

In conclusion our data demonstrate smaller oocyte diameters with increasing BMI, AMH and oocyte yield. These findings add to insights from animal models that describe the ovarian microenvironment as crucial for oocyte development and competence. They further emphasize the importance of more detailed oocyte assessments than are currently common practice, which may aid the currently used criteria for embryo selection and help to better understand how oocyte status is associated with later embryo development.

## Supporting information

S1 Table Effect of granular cytoplasm on oocyte and embryo characteristics. (DOCX)

S2 Table Effect of perivitelline space on oocyte and embryo characteristics. (DOCX)

S3 Table Anonymized raw data. (XLSX)
