# Supplementary material for: Age, body weight and ovarian function affect oocyte size and morphology in non-PCOS patients undergoing intracytoplasmic sperm injection (ICSI)
Source: PLoS One. 2019 Oct 24;14(10):e0222390. doi: 10.1371/journal.pone.0222390 (PMC6812759; doi:10.1371/journal.pone.0222390)
Supplement: S1 Table — (DOCX) [file pone.0222390.s001.docx]

|  | | | |
| --- | --- | --- | --- |
|  | No | Yes | p-value |
| *N* | 103 | 205 |  |
| *Total Oocyte Diameter* | 161.1 ± 7.0 | 162.7 ± 7.4 | 0.0698 |
| *Oolemmal Diameter* | 108.8 ± 3.4 | 109.6 ± 4.7 | 0.1634 |
| *2PN* | 76 (73.8%) | 128 (62.4%) | 0.0554 |
| *8 cells* | 39 (44.8%) | 65 (42.8%) | 0.7872 |
| *Good Embryo* | 43 (53.8%) | 77 (56.2%) | 0.7778 |
| *Embryo Used^[[1]](#endnote-1)^* | 54 (52.4%) | 125 (61.0%) | 0.1782 |

*S1 Table: Effect of granular cytoplasm on oocyte and embryo characteristics.*

1. [↑](#endnote-ref-1)
