# Supplementary material for: Age, body weight and ovarian function affect oocyte size and morphology in non-PCOS patients undergoing intracytoplasmic sperm injection (ICSI)
Source: PLoS One. 2019 Oct 24;14(10):e0222390. doi: 10.1371/journal.pone.0222390 (PMC6812759; doi:10.1371/journal.pone.0222390)
Supplement: S2 Table — (DOCX) [file pone.0222390.s002.docx]

| *S2 Table: Effect of perivitelline space on oocyte and embryo characteristics.* | | | |
| --- | --- | --- | --- |
|  | No | Yes | p-value |
| *N* | 264 | 44 |  |
| *Total Oocyte Diameter* | 161.4 ± 7.1 | 166.7 ± 6.7 | <0.0001 |
| *Oolemmal Diameter* | 109.6 ± 4.3 | 107.7 ± 3.8 | 0.0082 |
| *2PN* | 174 (65.9%) | 30 (68.2%) | 0.8640 |
| *8 cells* | 92 (44.4%) | 12 (37.5%) | 0.5664 |
| *Good Embryo* | 101 (54.6%) | 19 (59.4%) | 0.7018 |
| *Embryo Used* | 147 (55.7%) | 32 (72.7%) | 0.0467 |
